# Supplementary material for: Volumetric study of the maxillary sinus in patients with sinus pathology
Source: PLoS One. 2020 Jun 18;15(6):e0234915. doi: 10.1371/journal.pone.0234915 (PMC7302704; doi:10.1371/journal.pone.0234915)
Supplement: Attachment — Submitted filename: Letter point by point.doc [file pone.0234915.s001.doc]

May 17, 2020

Giovanni Cammaroto

Academic Editor

PLOS ONE

Dear Editor

We would like to submit the revised manuscript (PONE-D-20-10893**)** entitled “Volumetric study of the maxillary sinus in patients with sinus pathology” to the Editorial Board of PLOS ONE. All the changes have been highlighted in bold yellow along the manuscript.

We would like to thank the reviewers for their thoughtful review of the manuscript. We agree with the comments and we have revised our manuscript accordingly.

After completion of the suggested edits, the revised manuscript has benefitted from an improvement in the overall presentation and clarity.

Thank you very much for your attention and we look forward to hearing from you at your earliest convenience.

Sincerely Yours,

Prof. Dr. Mario Pérez-Sayáns

Journal requirements:

When submitting your revision, we need you to address these additional requirements:

1.    Please ensure that your manuscript meets PLOS ONE's style requirements, including those for file naming. The PLOS ONE style templates can be found at <http://www.plosone.org/attachments/PLOSOne_formatting_sample_main_body.pdf> and <http://www.plosone.org/attachments/PLOSOne_formatting_sample_title_authors_affiliations.pdf>

We have checked carefully all the documents and adapted the manuscript accordingly

2. Please note that according to our submission guidelines (<http://journals.plos.org/plosone/s/submission-guidelines>), outmoded terms and potentially stigmatizing labels should be changed to more current, acceptable terminology. For example: “Caucasian” should be changed to “white” or “of [Western] European descent” (as appropriate).

Outmoded terms have been changed to more current terminology.

3. In the Methods section please specify the date (month/year) when participants' medical records were reviewed.

Date has been added.

Reviewer #1: I thank the editor who gave me the opportunity to review this interesting article about the volumetric study of the maxillary sinus in patients with sinus pathology.
Introduction
(SNOT-22) [5] and Lund-Mackay [6] and Zinreich are clinical scores and not traditional techniques.

We thank the reviewer this consideration and it has been modified.

Statistical analysis
In which case did you use the chi-square test?

We have completed the explanation as follows: “Contingency tables were drawn up using the Chi-squared test to compare categorical variables”

Result
In Table 1, which statistical test did you use to compare the volumes of the sinuses (the ANOVA cannot be used) ???

Thank you very much for the appreciation. We used the t-student test for independent samples to compare means. Although for wide series we could assume the same p-value. This information has been added to material and methods section

In table 2, specify that you have written the Bonferroni correction only for the statistically significant values (p <0.05).

We appreciate this comment and we have added this information.

Discussion
You have written a lot about the use of CBCT for 3D measurement, I recommend adding these 2 articles that instead talk about the traditional CT system for navigation and 3D reconstruction, so you can do a comparison
Galletti B, Gazia F, Galletti C, Galletti F. Endoscopic treatment of a periorbital fat herniation caused by spontaneous solution of continuity of the papyracea lamina. BMJ Case Rep. 2019 Apr 30;12(4). pii: e229376.
Galletti B, Gazia F, Freni F, Sireci F, Galletti F. Endoscopic sinus surgery with and without computer assisted navigation: A retrospective study. Auris Nasus Larynx. 2019 Aug;46(4):520-525.
Another interesting article to mention about CBCT and the maxillary sinus is the following
Lo Giudice A, Galletti C, Gay-Escoda C, Leonardi R. CBCT assessment of radicular volume loss after rapid maxillary expansion: A systematic review. J Clin Exp Dent. 2018 May 1;10(5):e484-e494.

We thank the reviewer to these interesting papers. They have been added and accordingly cited in the discussion section in order to improve this specific section.

Basically the article says nothing new compared to that already present in the literature. The authors underline how the CBCT method, with particular 3D reconstruction software, can have economic advantages, compared to conventional techniques. I would give more emphasis to the conclusions, underlining that the CB method subjects the patient to a lower number of radiation than traditional CT, having the same advantages.

This is a good appreciation and we have added it to the conclusions

Reviewer #2: I found this manuscript appropriate, well designed and clearly exposed, for specialists and for non-specialists. Your results and discussion address the hypothesis and objectives, that are of interest in this field.
I suggest just to review a few details, or solve some doubts I have, to improve the final manuscript, as follows:

- I would appreciate if you clarify if the term "studied element" in the section of exclusion and inclusion and exclusion criteria, refers to the "Unit of Analysis". In this case, it could be a better term.

We are very sorry for the misunderstanding, of course the correct form is Unit of analysis and it has been changed accordingly.

- Could you specify the voxel size and FOV of the CBCT used?

Thank you very much for your comment, we have added this information in Material and Methods Section.

*Due to the deep anatomical and structural analysis of the radiological studies the values of FOV were high, from 170-229 mm. Regarding the voxel size used, it was between 0.3-0.4 as a general range, adapted to each specific case, so there was absolute precision when calculating the volume.*

- It's not clear enough how did you defined an affected sinus by dental pathology reason, that you finally excluded from the sample. I suggest just to clarify this point.

Due to the high and sometimes reversible morbidity, specific characteristics and also the behaviour of odontogenic sinus pathology we have excluded from this study lesions derived from radicular pathology: radicular cysts, periodontal or lateral cysts and other inflammatory lesions developed from a dental destruction/cavities or periodontitis.

- I suggest to include a comment in discussion about implications for future research in this field.

We appreciate this suggestion and we have added a comment addressed to this

- I find slightly contradictory the comment in discussion about the "reduced sample size" considering that your initial sample could be greater, and you reduced it by calculating a sample size properly.

Thank you very much for your comment, in fact it has been poorly expressed. We tried to compare with other bigger samples in terms of sinus pathology studies. We have deleted this comment.

- I suggest to write a final conclusion, that is optional, to clearly resume your findings if you find it possible and interesting.

A conclusion section has been added after the discussion by adding also specific suggestions from other reviewers who have given the same recommendation.

Thank you dear college.

While revising your submission, please upload your figure files to the Preflight Analysis and Conversion Engine (PACE) digital diagnostic tool, <https://pacev2.apexcovantage.com/>. PACE helps ensure that figures meet PLOS requirements. To use PACE, you must first register as a user. Registration is free. Then, login and navigate to the UPLOAD tab, where you will find detailed instructions on how to use the tool. If you encounter any issues or have any questions when using PACE, please email us at [figures@plos.org](mailto:figures@plos.org). Please note that Supporting Information files do not need this step.

We have uploaded the figures to PACE system and after the checking, they have been submitted with the reviewed version of the manuscript to the PLOS ONE system.
